# *Escherichia coli* Peptidoglycan Structure and Mechanics as Predicted by Atomic-Scale Simulations

**DOI:** 10.1371/journal.pcbi.1003475

**Published:** 2014-02-20

**Authors:** James C. Gumbart, Morgan Beeby, Grant J. Jensen, Benoît Roux

**Affiliations:** 1 School of Physics, Georgia Institute of Technology, Atlanta, Georgia, United States of America; 2 Imperial College London, South Kensington Campus, London, United Kingdom; 3 California Institute of Technology and Howard Hughes Medical Institute, Pasadena, California, United States of America; 4 Department of Biochemistry and Molecular Biology and Gordon Center for Integrative Science, The University of Chicago, Chicago, Illinois, United States of America; University of Notre Dame, United States of America

## Abstract

Bacteria face the challenging requirement to maintain their shape and avoid rupture due to the high internal turgor pressure, but simultaneously permit the import and export of nutrients, chemical signals, and virulence factors. The bacterial cell wall, a mesh-like structure composed of cross-linked strands of peptidoglycan, fulfills both needs by being semi-rigid, yet sufficiently porous to allow diffusion through it. How the mechanical properties of the cell wall are determined by the molecular features and the spatial arrangement of the relatively thin strands in the larger cellular-scale structure is not known. To examine this issue, we have developed and simulated atomic-scale models of *Escherichia coli* cell walls in a disordered circumferential arrangement. The cell-wall models are found to possess an anisotropic elasticity, as known experimentally, arising from the orthogonal orientation of the glycan strands and of the peptide cross-links. Other features such as thickness, pore size, and disorder are also found to generally agree with experiments, further supporting the disordered circumferential model of peptidoglycan. The validated constructs illustrate how mesoscopic structure and behavior emerge naturally from the underlying atomic-scale properties and, furthermore, demonstrate the ability of all-atom simulations to reproduce a range of macroscopic observables for extended polymer meshes.

## Author Summary

The structure of the bacterial cell wall has been a point of controversy and contention since it was first discovered. Although the basic chemical composition of peptidoglycan, the key constituent of the cell wall, is now well established, its long-range organization is not. This dearth of information at the mesoscopic scale is a result of the inability of experimental imaging techniques to simultaneously visualize both the atomic-level detail of the peptidoglycan network and its macroscopic arrangement around the bacterium. Now, using molecular dynamics (MD) simulations, we have carefully constructed and validated models of sections of the *Escherichia coli* cell wall in full atomic detail. By comparing various properties of these models, including elasticity, pore size, and thickness with experiments, we can discriminate between them, resolving which best represents the native wall structure. In doing so, our study provides approaches for connecting measurements made in atomic-scale MD simulations with large-scale and even macroscopic properties.

## Introduction

**Figure 1:**
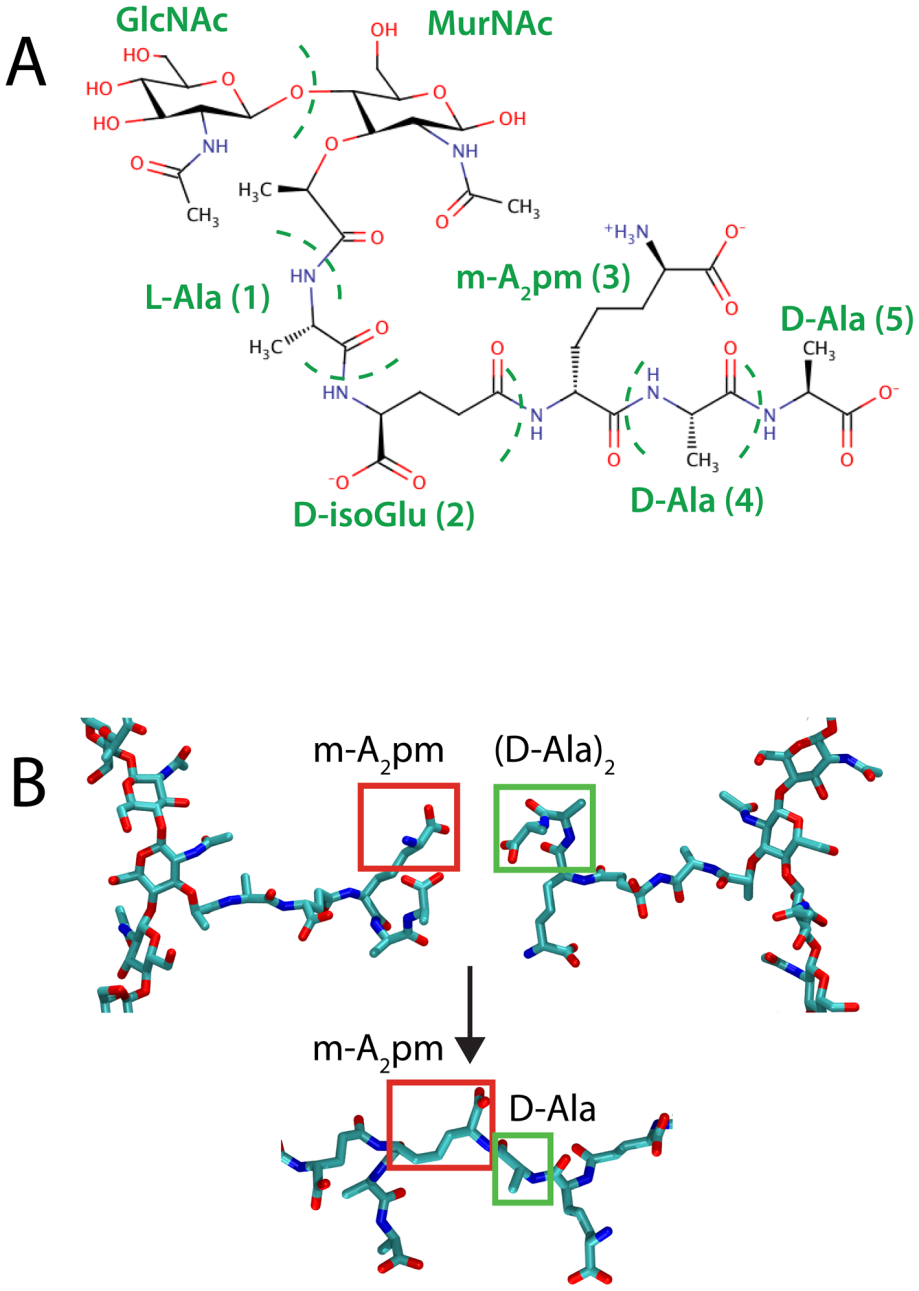
Peptidoglycan constituents. (A) Chemical composition of the monomeric unit of peptidoglycan, consisting of a disaccharide with a connected five-residue peptide. (B) Transpeptidation reaction between two neighboring peptidoglycan strands. The reacting groups contributed by each peptide are boxed in red and green, respectively, before and after being linked.

The cell wall rests outside the cytoplasmic membrane and provides bacteria with shape, rigidity, and protection from lysis due to the significant turgor pressure emanating from within [1]. It is primarily composed of a porous, mesh-like network of polymerized peptidoglycan, a repeating disaccharide/oligopeptide molecule. Because it is covalently connected, the cell wall is also the largest macromolecule in nature [2]. The chemical composition of peptidoglycan is largely conserved: relatively long glycan strands are cross-linked by short oligopeptides (see Fig. 1) [3]. In Gram-negative bacteria the cell wall presents as a relatively thin network (2–7 nm) between the inner and outer membranes, while in Gram-positive bacteria, it is much thicker, between 20 and 35 nm [1], [4].

Multiple theoretical models for the architecture of peptidoglycan at the mesoscopic scale have been conceived [1]. The models fall into two primary classes, a horizontal layer in which the glycan strands run parallel to the cell surface in a circumferential direction [5]–[7] and a scaffold in which the strands are oriented perpendicular to the surface [8], [9]. While some experiments have been interpreted as support for the scaffold model, e.g., the NMR structure of a peptidoglycan subunit [10], more recent electron cryo-tomography (ECT) on purified Gram-negative sacculi revealed circumferential glycan strands [11]. Even more complex models have been put forth, such as cables of coiled peptidoglycan encircling Gram-positive bacteria based on atomic force microscopy (AFM) measurements [12], although ECT on these bacteria failed to find distinct cable-like structures [4]. Using biochemical experiments and atomic-scale simulations, it was demonstrated that only layers composed of circumferential glycan strands could fully account for the ECT observations on Gram-positive bacteria, namely a distinct curling and thickening behavior of the sacculus, i.e., the part of the cell wall remaining after cell lysis, upon shearing [4].

One limitation of many of the previously developed models is that they are typically constructed with an idealized geometric arrangement, which is then deformed according to a set of mathematical rules. Not surprisingly, this procedure tends to generate cell walls with an unnatural degree of order [3]; indeed, regular patterns of quadrilateral or hexagonal shapes are often depicted [7], [8]. Furthermore, while apparent disagreement with a chosen set of experimental data has been used to indict some models over others [9], an alternative explanation is that the specific rules used to construct the model as well as the presumed experimental constraints were too strict [13].

In an attempt to circumvent some of the limitations of previous models, we have constructed and simulated patches of Gram-negative cell wall in their full atomic detail. By modeling the cell wall as a intricate composite of its individual components, we restrict the number of assumptions necessary for its construction. A single-layered model was chosen based on ECT of Gram-negative sacculi and on recent simulations of Gram-positive cell-wall patches [4]. Each patch of peptidoglycan was built from the level of individual residues on up, quantifying the behavior at each level, and connecting it with experimental measurements of various structural and mechanical properties. These comparisons are used both to validate the constructions and to illustrate the robustness of the cell wall to variations in average glycan strand length within the range of those observed in vivo. The widespread agreement with experimentally measured properties favors the disordered circumferential model of peptidoglycan in Gram-negative bacteria over other models.

## Results

### Modeling and validation of the physical properties of peptidoglycan components

While the glycan strand composition is uniform across all bacteria, composed of alternating 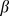-1,4-linked GlcNAc and MurNAc saccharides, the peptide stem, connected to the lactyl moiety of the MurNAc residue, is quite diverse [3], [6]. In *E. coli* the full, five-residue sequence is L-Ala (1) D-isoGlu (2) 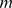-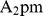 (3) D-Ala (4) D-Ala (5), where 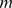-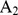
pm is 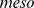-diaminopimelic acid, a lysine derivative [3]. Also of note is that the D-isoglutamate is connected through a 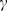-carboxy linkage to the 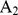
pm residue (see Fig. 1A). While alanine is already present in the CHARMM force field, the remaining four constituents were originally absent. Therefore, we developed new CHARMM-compatible topologies and parameters for these constituents, as well as for the connections between them (see Methods along with topology and parameter files provided in the Supporting Information). The novel force fields have already been successfully utilized for simulation of Gram-positive peptidoglycan [4].

**Figure 2:**
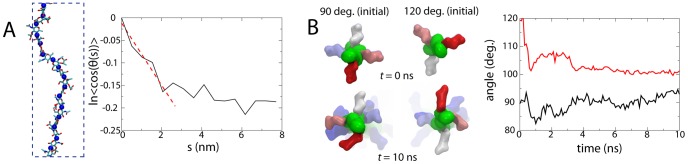
Properties of single glycan strands. (A) Calculation of the persistence length of the 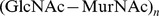 polysaccharide. Blue spheres along the strand on the left indicate the oxygen atoms involved in the glycosidic bonds between residues. (B) Average angle between neighboring peptides. On the left are images looking down on a portion of the strand (green) with the initial residues of the peptides shown, colored to indicate depth (red, then white, then blue). The plot on the right shows the average peptide-peptide angle as a function of time for one started at 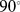 (black) and one started at 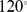 (red). Light restraints (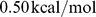
Å^2^) in the strand direction were placed on the glycosidic oxygen of every other GlcNAc residue to keep the strand elongated without preventing rotation. Also see Movie S1.

After parameterization, a single glycan strand 320 residues long was constructed without peptides. A useful property to quantitatively characterize the flexibility of a polymer is the persistence length 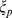. It is defined as
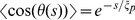
(1)where 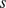 is the position along the strand, 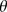 is the angle between the tangent vectors at positions 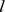 and 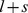, and the average is taken over all starting positions 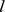 [14]. Effectively, 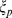 is a measure of the stiffness of the strand. Two 5-ns simulations of the 320-mer strand were carried out, and 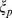 was determined by the initial decay of the correlation in Fig. 2 [15], [16]. The two simulations provided values of 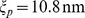 and 13.6 nm; extending the latter simulation to 10 ns changed this value only marginally (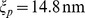). This persistence length is of the same order of magnitude as that found for other simple polysaccharides from experiments and/or modeling, which can span a large range, e.g., 4.5–13.5 nm for pectin [17] and 14.5 nm for cellulose [18]. Although not measured here, the persistence length of peptide cross-links is at least an order of magnitude less, being no more than 3–4 Å, making them significantly more stretchable than the relatively rigid glycan strands.

The peptides project outward from the glycan strand, presumably in a helical fashion (see Fig. 2B) [1]. The periodicity of these peptides is intimately connected to the orientation and degree with which neighboring glycan strands can form cross-links with one another. An angle of 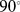 between successive peptide side chains was assumed in the classical layered model, thus placing every other one in the plane of the cell wall [5], [6]. An NMR structure of a peptidoglycan fragment, however, displayed an angle of 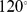, in line with that in the scaffold model [10]. To determine the equilibrium angle for an isolated peptidoglycan strand, two 60-residue-long strands were constructed and simulated for 10 ns, one with an initial peptide-peptide angle of 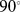 and one with an angle of 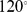. For the strand initially at 120, the average angle relaxed to 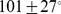 by the end of the 10-ns simulation, while the one initially at 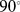 was 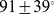 (see Fig. 2B). Based on these results, we conclude the native periodicity of the peptides is approximately four per turn. However, the significant variability in the angle in simulations, even within a single strand, indicates that this periodicity is not strictly maintained and could be easily modified by external forces.

### Mesoscale organization of the cell wall

In order to construct the full peptidoglycan network, individual glycan strands need to be covalently linked through their peptides. Although this linkage takes a variety of forms depending on species, in *E. coli* the most common link is a peptide bond made between the 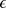 amino group of the 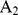
pm residue (position 3) and the carbonyl group of the penultimate D-Ala (position 4), shown in Fig. 1B [6]. In the course of transpeptidation, the terminal D-Ala (position 5) is also cleaved, both processes being carried out by penicillin-binding proteins [19]. The degree of cross-linking varies between species and even growth states within a single species [1]; for *E. coli* it is typically around 50% on average, i.e., about half of the peptides are linked and half are free [20]. Although alterations to the cross-linking fraction likely affects the mechanical and structural properties of the peptidoglycan network, this variable is not explored in the current study.

**Figure 3:**
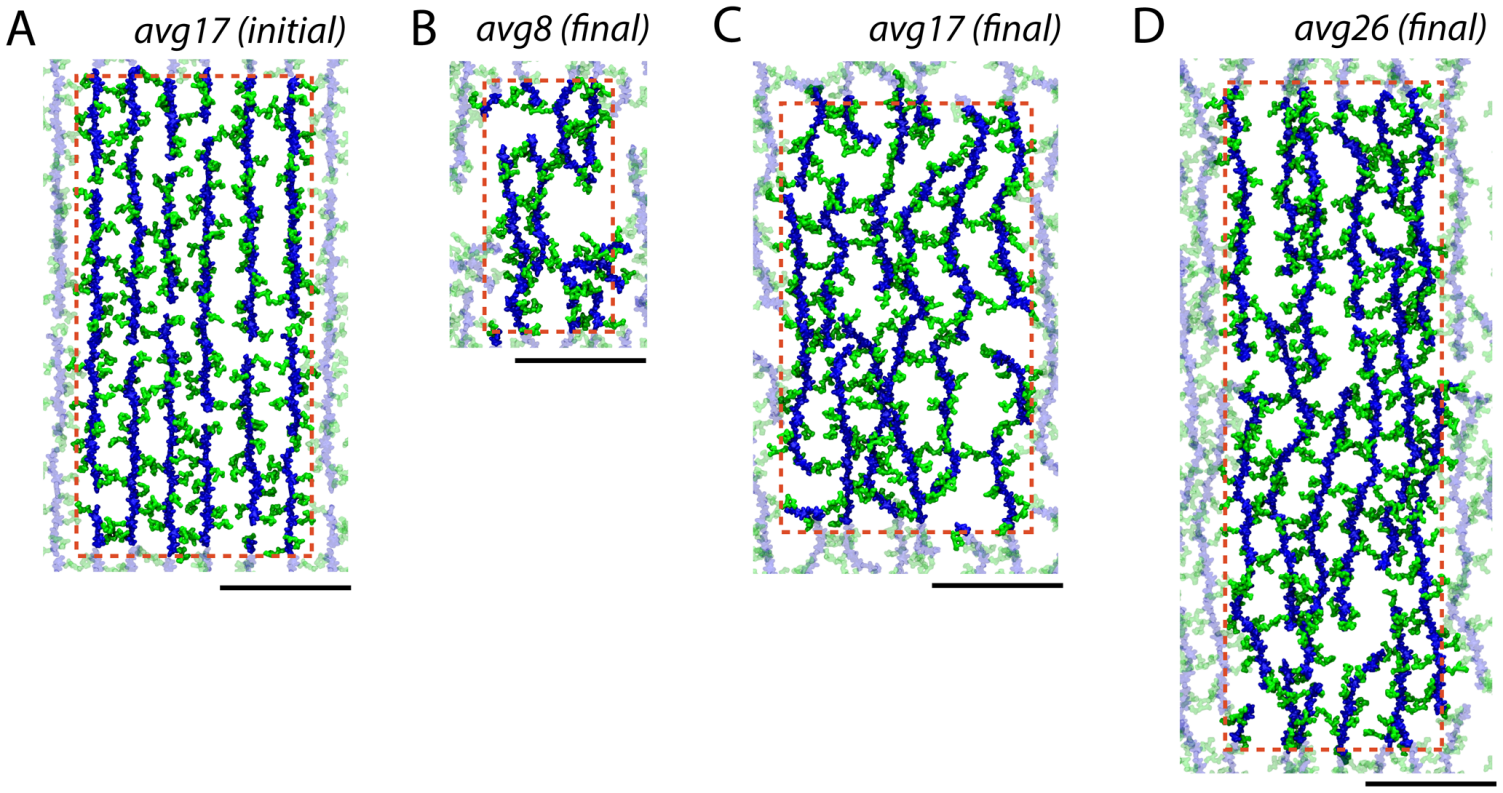
Peptidoglycan patches simulated. In all parts, glycan strands are in blue and peptides in green. The dotted red line denotes the unit cell boundaries, with the transparent peptidoglycan being periodic copies. The black scale bars below are all equivalent at 10(A) Initially constructed state for avg17 (other patches appeared similarly at this state). (B–D) Final relaxed states for (B) avg8, (C) avg17, and (D) avg26. Inf1 and Inf2 are shown in Fig. S6 in Supporting Information. Relaxation of avg17 is shown in Movie S2.

Two-dimensional periodic patches of peptidoglycan were constructed following a specific set of procedures designed to minimize user bias (see Methods); an example of a resulting system is shown in Fig. 3A. Despite being initially constructed as an organized, patterned network, the final organization of the peptidoglycan resembles the “disordered circumferential layered” model observed in cryo-tomography images of purified sacculi [11]. However, because no tension was applied, the possibility remains that the peptidoglycan becomes more ordered under native cellular conditions, which is explored below [1].

Much like the fraction of peptides cross-linked, in the bacterial cell wall the average glycan strand length takes on a large range of values, as low as six disaccharides in the stationary growth phase of *Helicobacter pylori* [21] and more than 50 in *Proteus morganii* [22]. Even in *E. coli*, a range of values spanning from 9 to 60 disaccharides has been measured by different experimental techniques for different stages of growth [1]. To examine the dependence of mesoscale properties of peptidoglycan on the average length of the glycan strand, multiple models with a specific average, but non-uniform, number of disaccharides were constructed, including 8
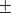
3.2, 17
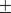
5.8, and 26
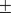
2.4 disaccharides, denoted avg8, avg17, and avg26, respectively (see Fig. 3). Additionally, as an extreme case for comparison, two patches of cell wall with unbroken, periodic (and therefore effectively infinite) glycan strands with unit-cell lengths of 15 and 30 disaccharides, denoted Inf1 and Inf2, were modeled. Because of the small number of strands used (12 for avg17 and avg26, for example) it's not possible to reproduce distributions, although the limited range of lengths in avg17 (8–26 disaccharides) does agree with where the majority of the strand lengths in CG models falls [23].

### Elastic properties of the peptidoglycan network

A defining property of the peptidoglycan layer is its tensile elasticity, i.e., its response to applied strain coming from the turgor pressure inside the bacterial cell, also referred to as Young's modulus. Elasticity also serves as a key metric for comparing the constructed models to experimental measurements. Because peptidoglycan is orthotropic, the elasticities along its two symmetry axes are not identical [24]. Based on the theory of mechanical deformation of a two-dimensional sheet (see SI for a full derivation starting from the material's constitutive relations), the Young's moduli in each orthogonal direction, 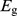 for the glycan strands and 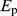 for the peptide cross-links, are given by
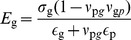
(2)

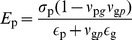
(3)where 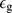 and 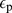 are the applied strains in each direction, defined as 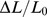, and 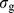 and 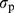 are the resulting stresses, measured in units of force/area. The dimensionless Poisson's ratios, 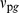 and 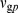, relate the spontaneous strain arising in one direction given an applied strain in the other. In order to calculate the elasticity from simulation, varying strains were applied in the plane of the peptidoglycan by altering its dimensions, with one dimension stretched and the other held fixed at its equilibrium value, calculated from a minimum 20-ns constant-pressure simulation (see Methods). Because only one of 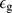 or 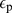 is allowed to be non-zero in each simulation, Eqs. 2 and 3 can be simplified to
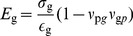
(4)

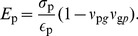
(5)While the stresses, 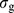 and 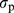, are formally the derivatives of the free energy with respect to strain in the corresponding dimension, by virtue of the reversible work theorem they can be directly related to the thermodynamic pressure in that dimension, i.e., a mean force (see Text S1) [25], [26].

Determination of each model's elasticity was based on six or more 2-ns-minimum simulations in which the peptidoglycan was stretched in the direction parallel to the glycan strands between 1.25% and 17.5% (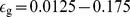) relative to the relaxed state and six more simulations between 5% and 45% (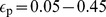) parallel to the peptide cross-links. Each simulation was repeated to ensure consistency of the results, giving at least 24 simulations per cell-wall patch (see Fig. S3 in SI). The resulting elasticities and Poisson's ratios are presented in Table 1, along with measurements and calculations from other studies [24], [27]–[30].

**Table 1:** Poisson's ratios (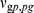) and Young's moduli 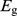 and 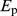 for simulated peptidoglycan patches compared with reported values from other studies.

Model	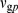	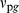	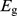 (MPa)	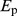 (MPa)	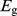 / 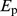
avg08	0.670	0.175	11.4	4.0	2.84
avg17	0.324	0.087	66.3	17.5	3.79
avg26	0.363	0.062	62.5	6.1	10.25
Inf1	0.216	0.020	125.7	11.3	11.05
Inf2	0.302	0.018	212.6	11.6	18.39
AFM [27]	0.48	0.16	45 (35–60)	25 (15–30)	1.17–4.0
exp. [29]	0	0	49±20	23±8.0	2.13
exp. [30]	0.4	0.4	-	50–150	-
theory [28]	-	-	-	30	-
theory [24]	0.35–0.67	0.03–0.23	10–32	3–5	2.0–10.7

**Figure 4:**
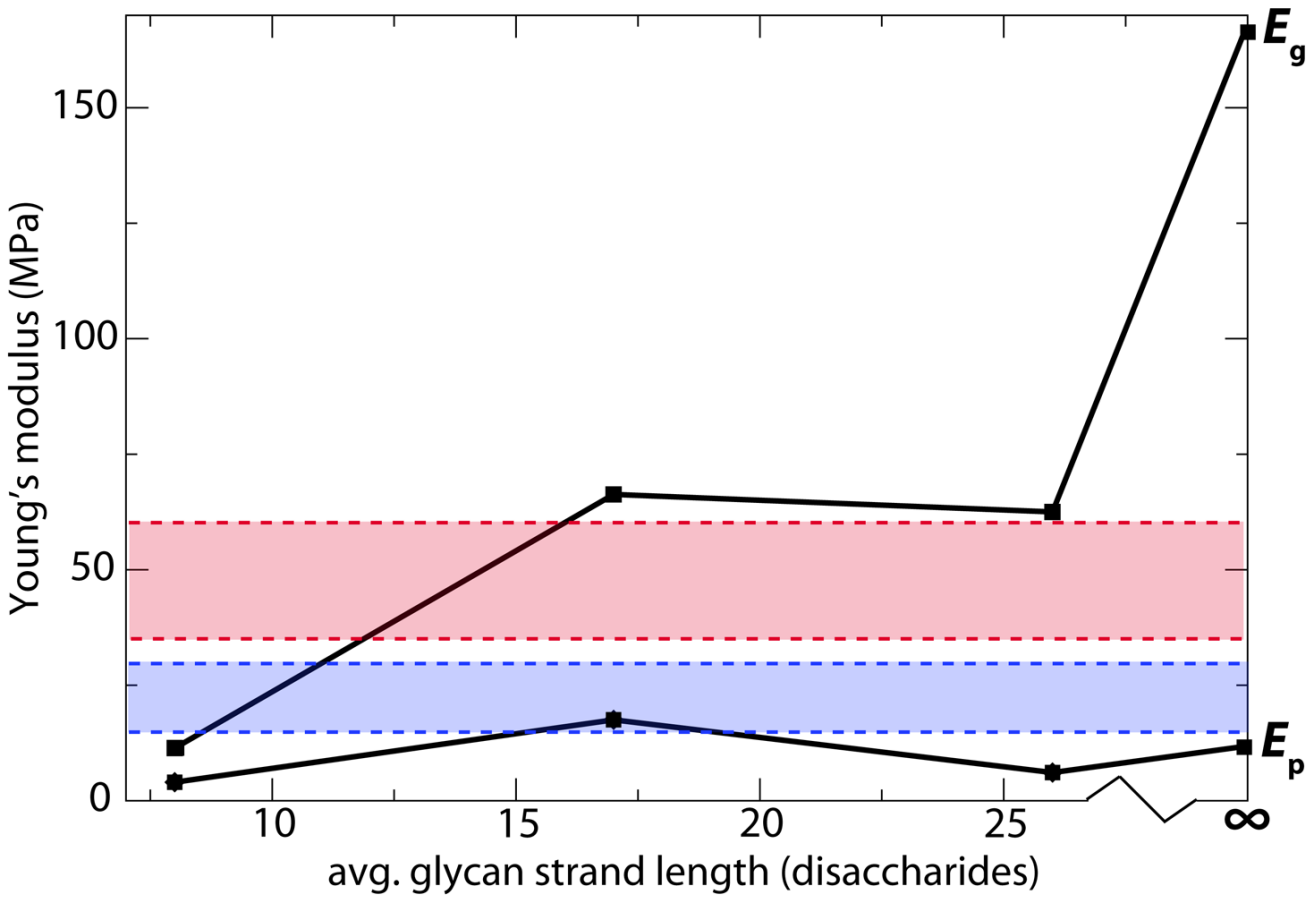
Young's modulus vs. average glycan strand length for the simulated patches. The elasticity in the glycan-strand direction is labeled 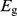 and that in the peptide direction is 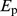. The red and blue bars represent the range of values measured in AFM experiments for the circumferential and axial cellular directions, respectively [27].

All simulated models reproduce the expected anisotropy of the elastic moduli in the two orthogonal directions [27]. The glycan strands are found to be much stiffer, with values of 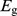 ranging from approximately 11 MPa to 66 MPa, compared to 4–18 MPa for 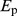 (both ranges for finite average strand lengths only). These values are similar to the ranges found in AFM measurements on *E. coli*, i.e., 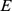 = 35–60 MPa perpendicular to the cell axis and 15–30 MPa parallel [27], as well as other theoretically derived elasticities [24], [29] (see Fig. 4). The glycan elasticity 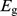 increases with average strand length, and for infinite strand lengths, it grows to as much as 200 MPa (see Inf1 and Inf2 in Table 1). 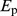, on the other hand, has no apparent correlation with average strand length.

The relationship between the Poisson's ratios, specifically that 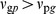 for all models, indicates that strain in the direction of the glycan strands induces a significant deformation in the peptide direction, but that the reverse is not true. Given that it is known that the glycan strands are aligned with the circumference of the cell and the peptide links with the long axis, the result is that stress applied to the cell wall will be primarily absorbed in the axial direction, leading to a lengthening of the cell, but not an increase in radius, just as observed experimentally [4], [31]. As average strand length increases, both Poisson's ratios decrease, effectively decoupling the two components of the peptidoglycan layer. One result of this decoupling is that the ratio of the two elasticities, 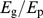, increases monotonically with average strand length.

### Quantifying macroscopic properties of the cell wall

Besides elasticity, other distinguishing physical characteristics of the cell wall as a whole include its thickness, the size of pores within it, the ordering of its strands, and the area per disaccharide. Using the five model patches developed, all of these characteristics were determined under different applied strains and compared to experimental measurements (see Table 2).

**Table 2:** Various properties of the simulated peptidoglycan patches under different applied strains.

Model	Applied strain	thick (dens.)	thick (stress)	angle (°)	Pore rad.	unit area ( 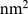 )
avg8	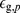 = 0	3.87	2.72	−7.8 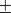 28.3	2.54	2.96
avg8	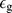 = 0.10	3.39	2.06	−3.8 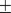 32.5	2.95	3.26
avg8	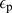 = 0.30	3.28	1.74	−3.8 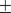 29.5	3.10	3.85
avg17	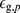 = 0	3.42	2.27	−2.9 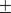 23.9	2.05	3.07
avg17	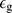 = 0.075	3.17	2.19	−3.0 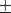 21.9	2.34	3.30
avg17	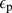 = 0.30	3.01	1.85	−2.2 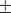 26.0	3.28	3.99
avg26	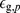 = 0	3.53	2.50	−0.6 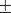 22.7	2.09	2.82
avg26	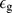 = 0.075	3.25	2.27	−0.4 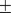 19.6	2.29	3.03
avg26	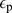 = 0.30	3.07	2.34	0.4 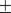 22.8	3.43	3.67
Inf1	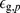 = 0	3.39	2.64	−1.5 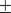 20.9	2.05	2.82
Inf1	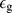 = 0.075	2.87	2.61	−0.9 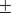 12.4	1.89	3.03
Inf1	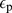 = 0.30	2.63	1.74	−1.3 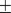 21.7	2.92	3.63
Inf2	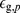 = 0	3.55	2.86	−0.3 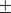 21.6	1.92	2.55
Inf2	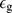 = 0.075	3.13	2.50	0.0 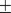 12.6	1.87	2.74
Inf2	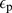 = 0.30	3.31	2.26	−0.1 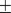 21.9	2.44	3.32

All thicknesses and radii are presented in units of nm. The most recent experiments have assigned a thickness of 2–4 nm at most [11], [34]. Pore sizes measured experimentally range from 2–3 nm in radius [5], [35] and even up to 5 nm in AFM experiments [34]. The experimental unit surface area is estimated to be 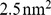 [37].

Based on different techniques, the thickness of the *E. coli* peptidoglycan layer has been assigned a range of values, including 2.5 nm (small-angle neutron scattering [32]), 6.0 nm (AFM [27]), and 6.4 nm (cryo-electron microscopy [33]). More recent ECT experiments estimated the thickness to be 4 nm at most [20] and AFM experiments measured ∼2 nm [34]. In contrast, cell walls from *Pseudomonas aeruginosa* appear even thinner, ranging from 2.4 nm (cryo-EM [33]) to 3.0 nm (AFM [27]), while that from *Caulobacter crescentus* is up to 7-nm thick [11].

**Figure 5:**
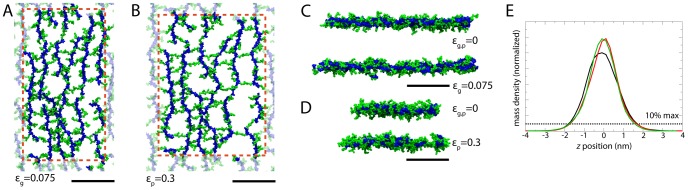
Peptidoglycan under strain. Shown in all panels is the avg17 cell-wall patch. The black bars in A–D are all 10 nm in length. (A,B) Top view with (A) 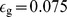 and (B) 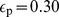. (C) Axial view (glycan strands oriented in the plane of the page). (D) Circumferential view (glycan strains pointing into the page). (E) Normalized mass density for 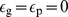 (black), 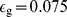 (red), and 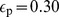 (green). Thickness was taken as the width at 10% of the maximum for each curve separately. A movie of stretching for 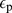 = 0.20 is also provided (Movie S3).

The thickness in the simulated constructs was determined in two different ways. First, the mass density as a function of 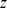, the coordinate orthogonal to the plane of the cell wall, was measured. This density was calculated for all the heavy atoms in the peptidoglycan, averaged over each trajectory. Values for the thickness were taken as the width of the density profile at 10% of its peak (see Fig. 5E). For the relaxed cell walls, the thickness ranged from 3.4–3.9 nm, in agreement with the most recent ECT measurements [11]. Under strain, this thickness decreased by up to 20%.

As a complementary measure of thickness, pressure profiles as a function of 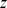 were measured for the patch in each simulation. The thickness was then taken to be the stress-bearing part of the wall, i.e., that fraction of the simulation system with a significantly increased pressure compared to bulk water (see Fig. S1 in SI). This thickness was as much as 1–1.5 nm less than that derived from the mass density. Such a result is unsurprising, as the free peptide chains, which project outward from the cell wall, will contribute to the mass density but do not bear any stress (see SI for a detailed discussion of the pressure profile calculations).

The maximum pore size in the cell wall in different states has been measured indirectly by determining the largest objects that can pass through it. For example, fluorescently labeled dextran molecules were used to estimate the pore radius in the *E. coli* wall as 2.06 nm in the relaxed state [5]. In another study, proteins up to 100 kDa in size were released from osmotically shocked cells, giving an estimated pore radius of 3.1 nm for stretched peptidoglycan [35]. For the cell walls with finite glycan strand lengths studied here, i.e., avg8, avg17, and avg26, the maximum pore radius averaged over time was 2.05 to 2.44 nm in the relaxed state (see Fig. S4). This radius almost uniformly increased under strain, with the maximum observed being 3.43 nm, in agreement with the large pore size observed in the osmotic-shock experiments [35]. These pore sizes fall in the same range as those observed in CG simulations [23], although neither captures the very large pores (10-nm diameter) observed in recent AFM experiments [34], most likely explained by cell wall-spanning macromolecular machinery [36].

ECT images of frozen *E. coli* sacculi have revealed a lack of significant ordering of the glycan strands in the relaxed state [11], although some question remains as to whether this disorder persists when the cell wall is under tension as in a living cell [1]. For the simulated cell walls, the ordering was quantified by measuring the angle between segments of each strand and the circumferential axis (see Fig. S7). The temporally and spatially averaged angle was typically close to 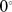 as expected, although persistently off-axis. The standard deviation in the angle was found to be significant at around 20–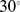 (see Table 2). For avg8, avg17, and avg26, the difference in this deviation was minimal under strain when compared to the relaxed state, suggesting that the wall does not become more ordered under tension. In contrast, for constructs Inf1 and Inf2, tension in the direction of the strands notably decreases their off-axis fluctuations. The sensitivity of these fluctuations to tension is due to the strands' inability to redirect the applied stress into the peptide cross-links, reflected also in their reduced Poisson's ratios (see Table 1).

The average surface area per disaccharide has been estimated at 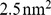 based on the number of 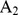
pm molecules in a given bacterium [37]. We note that this area can depend on a variety of factors, however, including ion concentration, pH, and the presence of denaturants [2], [4], although in the current study, only neutralizing 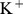 ions are used (see Methods). For the different patches examined here, the unit area for the relaxed cell wall ranges from 2.6 to 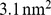, and rises to 3–4 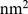 under strain (see Table 2). While at first, the discrepancy between experiment and modeling appears large, it should be noted that the cell wall may not be uniformly single layered, with up to three layers in some regions predicted [32]. Peptidoglycan in these additional layers would serve to lower the effective unit area for a single-layered cell wall. Considering a range of possible cell walls from completely single-layered to completely double-layered gives a range of possible unit areas of 2.5–5.0 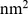. If one assumes that the initial strand spacing used during modeling is linearly related to the resulting unit area, this implies that the average strand spacing can be no less than 2 nm (unit area of 2–2.67 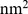) and no more than 4 nm (unit area of 4–5.33 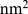).

## Discussion

The native architecture and organization of the bacterial cell wall are largely inaccessible to direct imaging techniques, though at the very edge of the resolution of ECT, glycan strands could be discerned in a Gram-negative sacculus. These strands were, nonetheless, fragmented, and the cross-links were indiscernible [11]. Furthermore, the imaged samples are no longer part of living cells. For these reasons, modeling fills a critical gap between biochemical data on the cell wall's constituents and biophysical data on its macroscopic properties. In this paper, patches of an *E. coli* cell wall were made using a circumferential layered model, supported by ECT imaging of both Gram-negative and Gram-positive sacculi [4], [11], plausibility arguments based on the thickness and glycan strand length [1], and the average peptide-peptide angle measured above (see Fig. 2B). The patches were constructed using only a few initial parameters, including the initial strand spacing (roughly 3 nm), degree of cross-linking (50%), and average glycan strand length (between 8 and 26 disaccharides).

In the simulated cell-wall patches, peptidoglycan was found to be relatively inelastic in the direction of the glycan strands, while very elastic in the direction of the peptide cross-links. The calculated Young's moduli for the two directions, 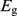 and 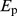, respectively, were found to be in good agreement with multiple AFM measurements [27], [29], with the best agreement being found for the constructs avg17 and avg26 (see Table 1). The average glycan strand lengths for these two constructs also match those measured experimentally for *E. coli* cells in the stationary (17.8) and exponential growth (25.8) phases [20]. 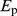 determined for live bacteria grown in aragose gel (5–15 MPa) was notably higher than our calculations, although other factors such as the outer membrane stiffness or incomplete gel polymerization may have inflated the number [30].

Further evidence of the relative stretchability of the peptide cross-links compared to the glycan strands comes from the decrease in Poisson's ratios as average glycan strand length increases. At short lengths, there is a significant coupling between the peptide cross-links and the glycan strands, allowing the former to absorb stress from the latter. At longer lengths, however, strain applied to the glycan strands is primarily absorbed by the strands alone, which, due to their inability to stretch much beyond their initial lengths, induces a large stress in the cell wall in their direction. This resistance to expansion, thus, does not depend on the peptide cross-links but is intrinsic to the glycan strands. Indeed, an intriguing suggestion is that longer glycan strands can compensate for a decrease in cross-linking percentage to maintain cell integrity [38]. While the fraction of peptides in cross-links was fixed near 50% for all models here, 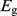 is directly related to the glycan strand length, whereas 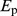 is independent. Although it remains to be shown, we hypothesize that, conversely, 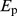 will be more sensitive than 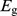 to the degree of cross-linking.

Beyond elasticity several other quantifiable properties were measured from the simulations, including the cell-wall thickness, maximum pore radius, and unit area per disaccharide. Excellent agreement with experimentally determined thicknesses [11] and pore sizes [5], [35] was found. The unit area measured in simulations (2.6–4 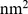) implies a cell wall that is more sparse than that estimated from experiment (2.5 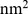). However, those experimental estimates are based on quantifying the total number of 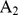
pm molecules per cell, irrespective of their place in the cell wall [37]. Neutron-scattering experiments have led to the suggestion that the Gram-negative cell wall is primarily a single layer, but includes regions of up to three layers over 25% of the surface [32]. The excess peptidoglycan in these additional, but limited and incomplete, layers would raise the experimental unit area for a single layer to 3.75 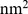, in significantly better agreement with that from the models examined here, also supporting the choice of initial strand spacing of 3 nm.

The average angle of the glycan strands with respect to the circumferential axis was found to be near 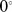, although the standard deviation was typically 20–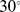, even under tension (see Table 2). The lack of alignment amongst the strands argues in favor of a disordered circumferential model, as previously indicated by ECT [11]. A chiral patterning of peptidoglycan has been suggested based on recent experiments, and was attributed to a helical movement of MreB, a proposed cytoskeletal protein [39], [40]. Recent total internal reflection fluorescence and ECT experiments have indicated, however, that MreB moves circumferentially around the cell and does not form long filaments [41]–[44]. The glycan-strand angle measured here was often negative (range of −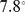 to 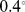), which hints at a slight intrinsic chirality in stressed peptidoglycan networks, irrespective of their assembly. Whether this could explain the experimental results remains unclear.

The widespread agreement between simulation and experiment for all of the aforementioned properties, including elasticity, thickness, pore size, and unit area, serves to validate the connection made between the modeled atomic-scale properties of peptidoglycan and the macro-scale properties probed experimentally. The present molecular models support a cell wall composed predominantly of a single layer of peptidoglycan with glycan strands running circumferentially around the cell in a disordered fashion. Furthermore, assuming our model is correct, we predict that the disorder, which is primarily due to the random orientation of the peptide cross-links relative to the strands, persists under native cellular conditions. While we do not consider possible growth mechanisms here in detail, the insertion of new peptidoglycan strands has been predicted to be a function of such disorder, as well as mechanical tension and MreB [45], [46]. To examine tension-dependent insertion, we also created a peptidoglycan patch in which one strand was deleted, tension applied, and then the strand was added back to the gap that formed. Because some cross-links between the re-added strand and the rest of the patch formed in alternate locations, a slight decrease in the degree of connectivity resulted and one larger pore was observed (radius of 3.6 nm vs. 2.9 nm; see Text S1 and Fig. S5 for more details). Because this pore may serve as a site for addition of the next peptidoglycan strand, it cannot be assumed that larger pores are an inevitable product of tension-dependent insertion. However, over repeated growth cycles, the insertion mechanism used is likely to become increasingly relevant to the large-scale structure that develops.

While a number of other simulations of bacterial cell walls have been carried out in recent years [23],[39],[47], they are all highly coarse grained (CG), a necessary approach for modeling complete sacculi. Coarse graining the system requires, however, that one make a number of assumptions about the properties of individual “beads” in the CG model, including what underlying atoms they represent, how they are connected and interact with each other, and how they are affected by the surrounding environment, e.g., solvent. Where possible such assumptions are rooted in experimental data, although the reliability of those data and their conversion to model parameters is not always straightforward. On the other hand, models built starting from the atomic scale, in which the parameters are not specialized for each application, can utilize the same experimental data for validation, as done here. The atomic-scale model is limited in size compared to the CG models, however, and therefore cannot fully reproduce distributions in strand length [23] nor capture structural features beyond the modeled scale, e.g., pore sizes up to 10-nm in diameter [34]; additionally, a visual comparison of the previous CG models with the atomic-scale models here suggests that the latter models are still too ordered, likely a remnant of the initial construction [23]. Thus, future iterations will be used to probe more realistic growth models, the dependence of cellular-scale properties on the cross-linking fraction and strand spacing, and also the interactions of the network with various growth and remodeling enzymes and embedded proteins.

## Methods

### Parametrization of novel residues and linkages

Force-field parameters for GlcNAc were developed by linking glucose and acetamide, with those charges and parameters near the interface determined. Similarly, MurNAc parameters were developed by linking GlcNAc with lactic acid. Charges of interfacial atoms, namely C2 on the sugar ring and the the NH group on the acetamide side chain in both residues along with C3 on the ring, the 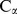 in the lactic acid side chain, and the bridging O3 oxygen in MurNAc, were modified. These charges were determined from ab initio quantum chemical calculations using a pre-release version of the Force Field Toolkit (ffTK) plugin for VMD, following the CHARMM parametrization procedures [48], [49]. Bond, angle, and dihedral parameters involving the interfacial atoms were similarly determined. Because D-isoglutamate and 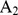
pm are nearly identical to their standard amino-acid counterparts, glutamate and lysine, their parameters were developed solely by analogy. The complete topology and parameter set used for subsequent simulations is provided in Text S2 and S3.

### System construction

Because simulating the actual transpeptidase reactions is prohibited by both current knowledge of the order of events and available computational resources, a procedure was developed to build the peptidoglycan network with a statistical view of the general organization. In the first step, a set of *E. coli* peptidoglycan strands with the number of disaccharides chosen according to a random Gaussian distribution of specified mean are placed parallel to one another separated by a given distance (typically 2–3 nm, with a 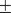
0.5 nm random deviation). Each system is fully solvated in explicit water and sufficient 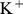 ions were added to the solution to neutralize the high negative charge in the peptidoglycan. The final atom count ranged from 100,000 to 545,000 atoms. Initially, the glycan strands are held fixed for a 2-ns simulation while the peptides are left free to move. Next, the trajectory is analyzed to find when each available 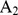
pm 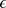-nitrogen first comes near an available D-Ala carbonyl oxygen, and for what fraction of time they are within this distance. Finally, the list of possible links is ordered according to the first contact using a more stringent distance criterion along with a minimum time within range. Links are then added, in order, such that when a given 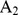
pm or D-Ala residue is linked, its entire peptide is removed from further consideration. The time and distance criteria are chosen to target roughly 50% cross-linking overall, as typically observed for *E. coli* [1], [20].

The cross-linked peptidoglycan network is first relaxed using energy minimization, and then allowed to equilibrate during MD simulations with no applied restraints. It should be noted that the network is periodic, with glycan strands as well as peptides covalently linked across the simulation system's periodic boundaries, thus mimicking a much larger patch of cell wall (see Fig. 3). The resulting network is simulated for at least 20 ns under constant pressure conditions, which allows its dimensions to fluctuate. The relaxed in-plane dimensions of each patch were taken as the average over the last 10 ns. These dimensions are: 9.3
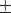
0.2
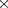
18.1
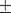
0.5 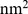 (avg8), 18.7
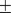
0.2
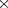
33.4
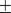
0.25 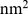 (avg17), 17.2
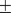
0.4
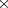
51.2
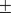
0.4 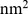 (avg26), 18.6
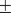
0.3
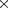
13.6
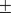
0.1 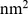 (Inf1), and 16.8
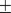
0.3
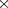
27.4
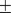
0.2 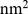 (Inf2).

### Molecular dynamics simulations

All simulations were run with the molecular dynamics package NAMD 2.9 [50] and the CHARMM force field [51]–[53]. A constant temperature of 310 K was held using Langevin dynamics; a pressure of 1 atm in the direction normal to peptidoglycan layer was maintained with a Langevin piston [54]. A 2-fs time step was utilized, with short-range non-bonded interactions (12-Å cutoff) evaluated every time step and long-range electrostatics every two time steps using the particle-mesh Ewald method [55]. All figures were made using VMD [56].

## Supporting Information

Figure S1Pressure profile along the glycan axis for simulation of avg17 patch with 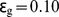. The grey line is the original profile computed in 1-Å slabs, with the black curve representing a 5-Å running average. The red line is the stress-bearing thickness of the peptidoglycan at 10% of the peak stress.(PNG)

Figure S2Pressure profiles along the 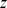 axis (normal to the peptidoglycan layer) for avg17 with 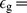
0.025, 0.05, 0.075, 0.1, 0.125, 0.15, and 0.175.(PNG)

Figure S3Stress as a function of strain for all simulated systems. In each plot, the black circles are data from simulations in which 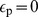 and 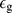 was varied, while the red squares are from simulations in which 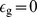 and 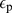 was varied. The corresponding lines are linear fits to the data.(PNG)

Figure S4 Patch of cell wall with maximum-radius spheres inscribed. Unlike in other figures, here the glycan strands are in grey and the peptides in tan. Sphere color is assigned based on size, with blue representing those with radius less than 1 nm, green less than 1.25 nm, yellow less than 1.5 nm, orange less than 1.75 nm, and red greater than 1.75 nm.
(PNG)

Figure S5Strain-dependent insertion. In both panels, the avg17 patch is under strain 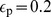. Glycan strands are in blue and peptide cross-links in green. The strand selected for deletion and later replacement is shown in red and orange. (A) Original patch. (B) Patch after strand deletion, equilibration, and subsequent strand replacement.(PNG)

Figure S6 Peptidoglycan patches simulated with effectively infinite strand lengths, colored as in Fig. 3 in the main text. The black scale bars below are all equivalent at 10 nm in length. Final relaxed states for (A) inf15 and (B) inf30 are shown.
(PNG)

Figure S7 Quantifying glycan-strand angle as a measure of disorder. Shown are the NAG and NAM saccharide rings against a transparent outline of the full cell wall viewed from the outside. Individual angles made with the dashed line were measured for all vectors connecting the centers of rings spaced at least four saccharides apart, although only a subset of vectors are shown here. These vectors were then averaged over all separations within a given strand, over all strands within the simulated cell-wall patch, and over all frames in the simulation trajectory. The black, red, green, and purple vectors give positive angles, while the blue vector gives a negative angle. The dashed line represents the cell's circumferential axis with which the glycan strands were initially aligned during construction.
(PNG)

Movie S1 Simulation of a 320-mer glycan strand for 5 ns.
(MPG)

Movie S2 Relaxation of the avg17 patch after cross-linking of the peptides.
(MPG)

Movie S3 Response of the avg17 patch after a strain of 0.2 is applied in the peptide direction.
(MPG)

Text S1 Formal derivation of the stress-strain relationships used in the study, expanded methods for measurements, and a discussion of simulations of strain-dependent strand insertion.
(PDF)

Text S2 CHARMM-force field formatted topology file for the residues unique to this study.
(TXT)

Text S3 CHARMM-force field formatted parameter file for the residues unique to this study.
(TXT)
