# Supplementary figures and images for: Escherichia coli Peptidoglycan Structure and Mechanics as Predicted by Atomic-Scale Simulations
Source: PLoS Comput Biol. 2014 Feb 20;10(2):e1003475. doi: 10.1371/journal.pcbi.1003475 (PMC3930494; doi:10.1371/journal.pcbi.1003475)

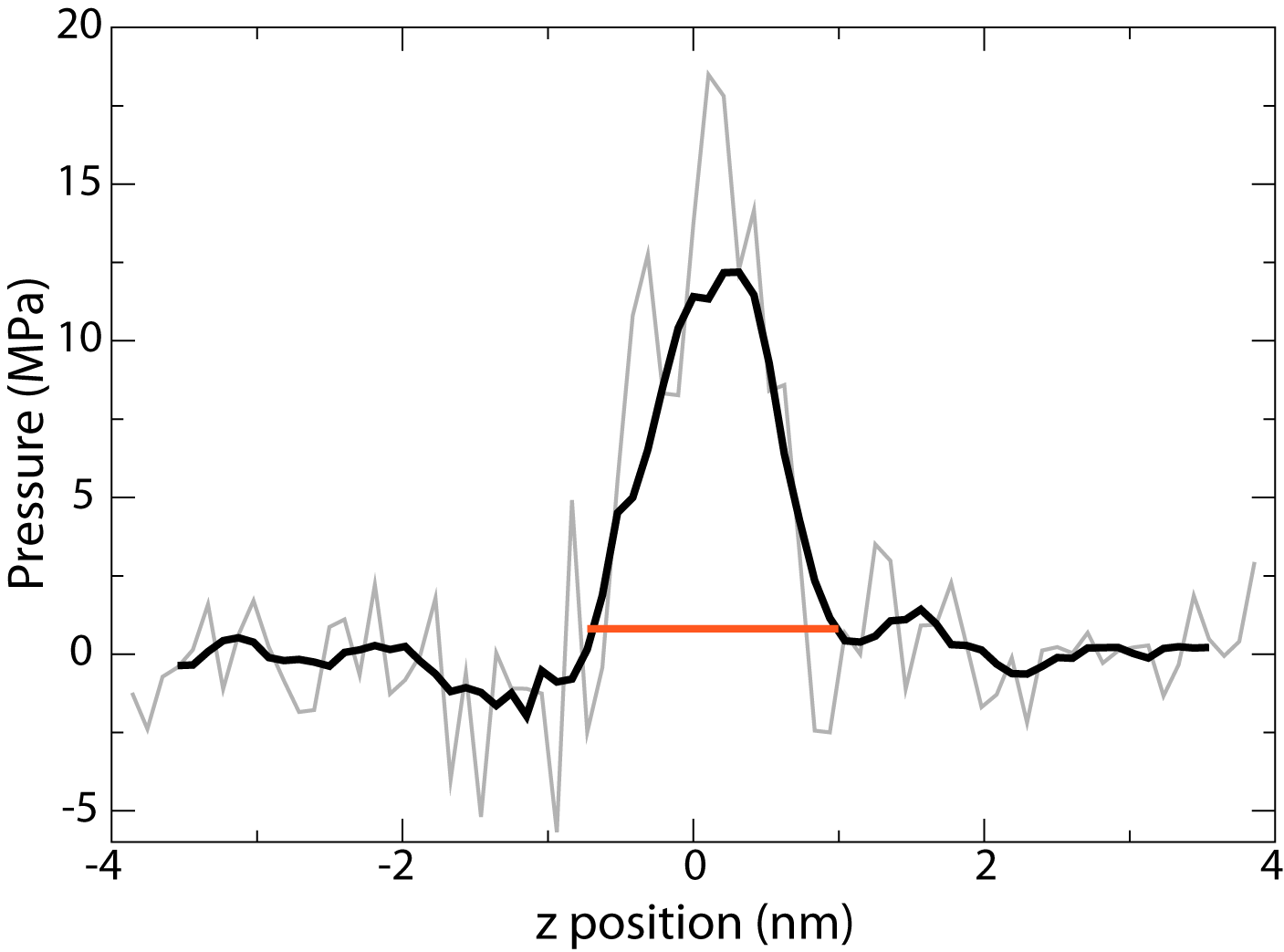

Supplement: Figure S1 — Pressure profile along the glycan axis for simulation of avg17 patch with . The grey line is the original profile computed in 1-Å slabs, with the black curve representing a 5-Å running average. The red line is the stress-bearing thickness of the peptidoglycan at 10% of the peak stress. (PNG) [file pcbi.1003475.s001.png]

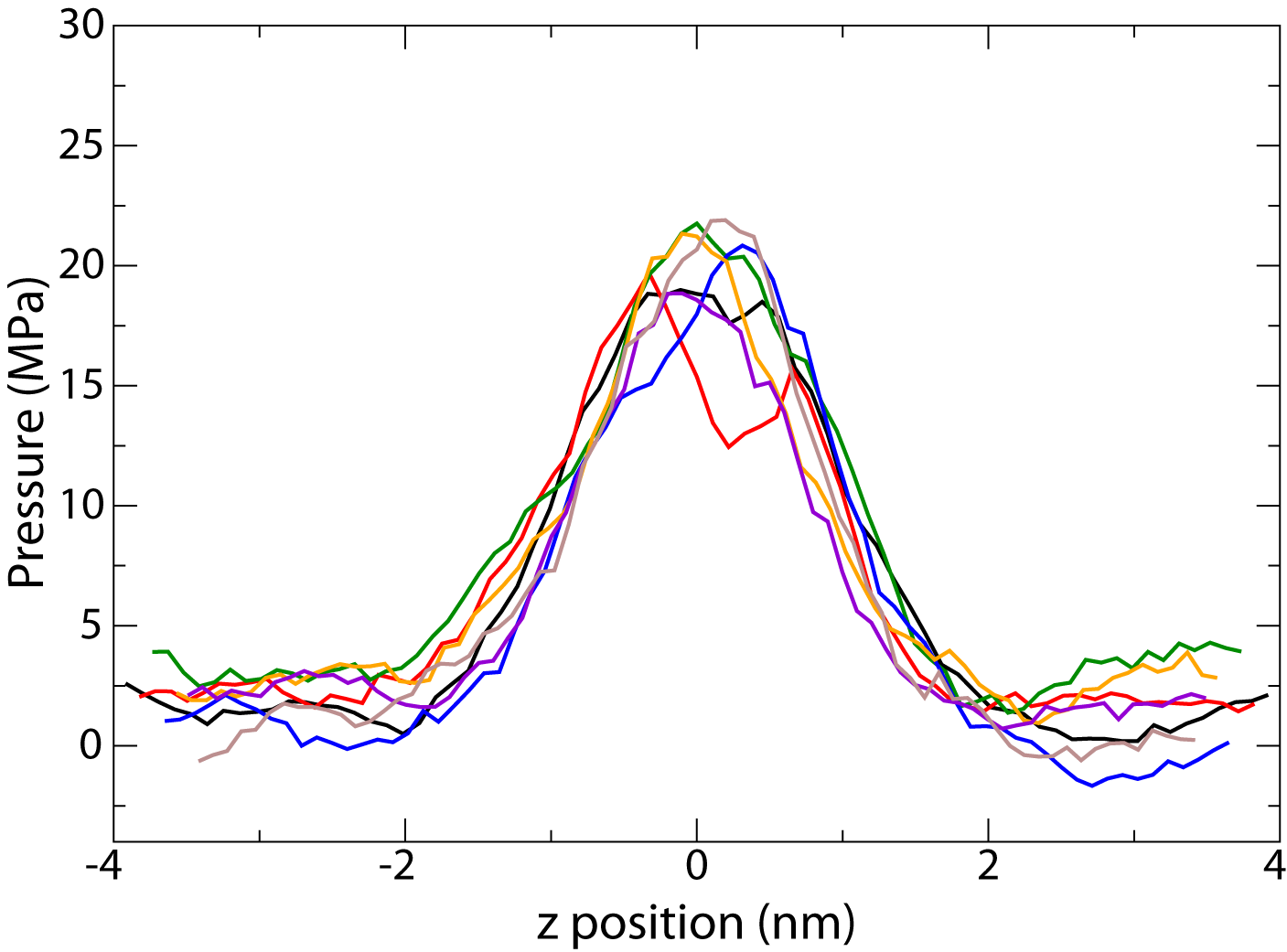

Supplement: Figure S2 — Pressure profiles along the axis (normal to the peptidoglycan layer) for avg17 with 0.025, 0.05, 0.075, 0.1, 0.125, 0.15, and 0.175. (PNG) [file pcbi.1003475.s002.png]

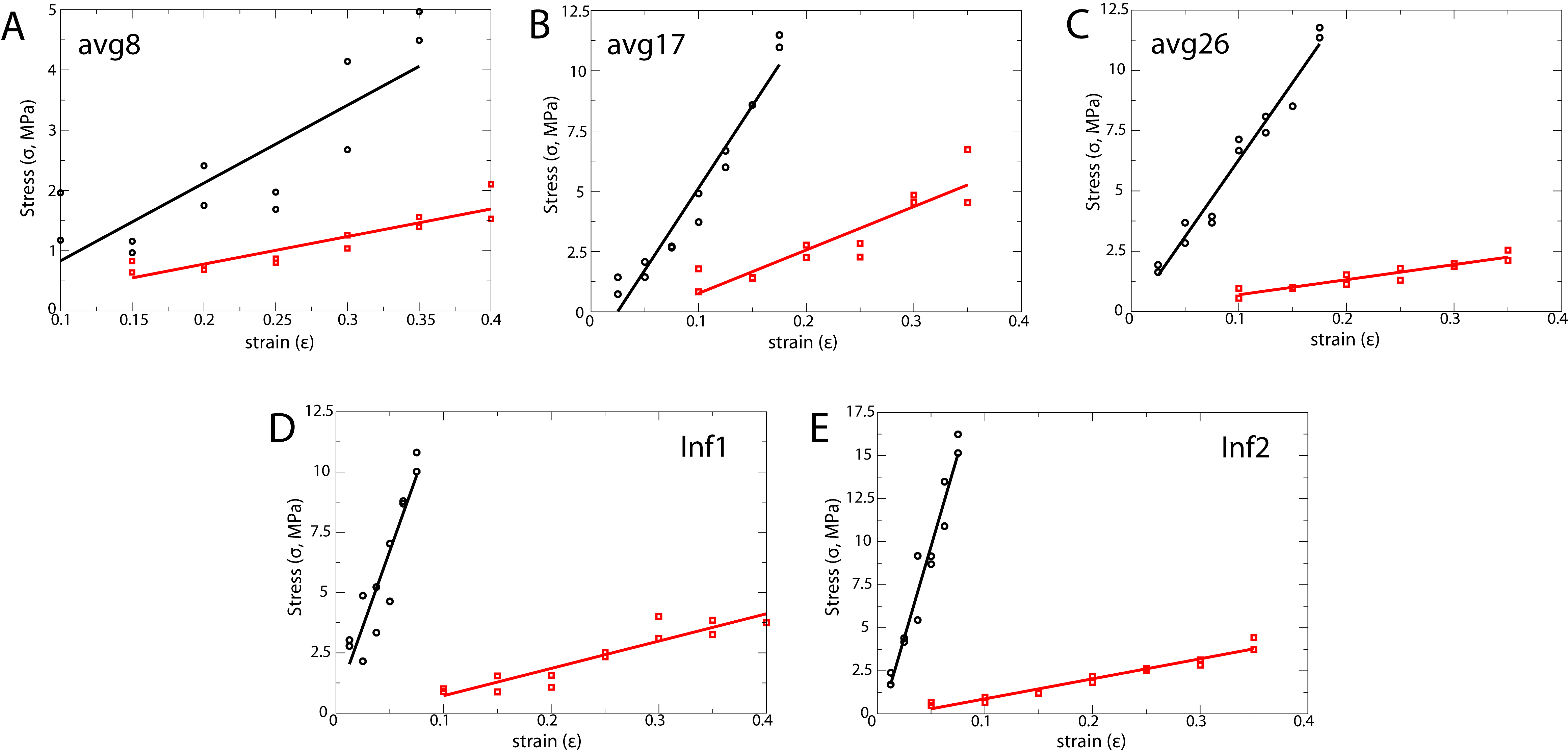

Supplement: Figure S3 — Stress as a function of strain for all simulated systems. In each plot, the black circles are data from simulations in which and was varied, while the red squares are from simulations in which and was varied. The corresponding lines are linear fits to the data. (PNG) [file pcbi.1003475.s003.png]

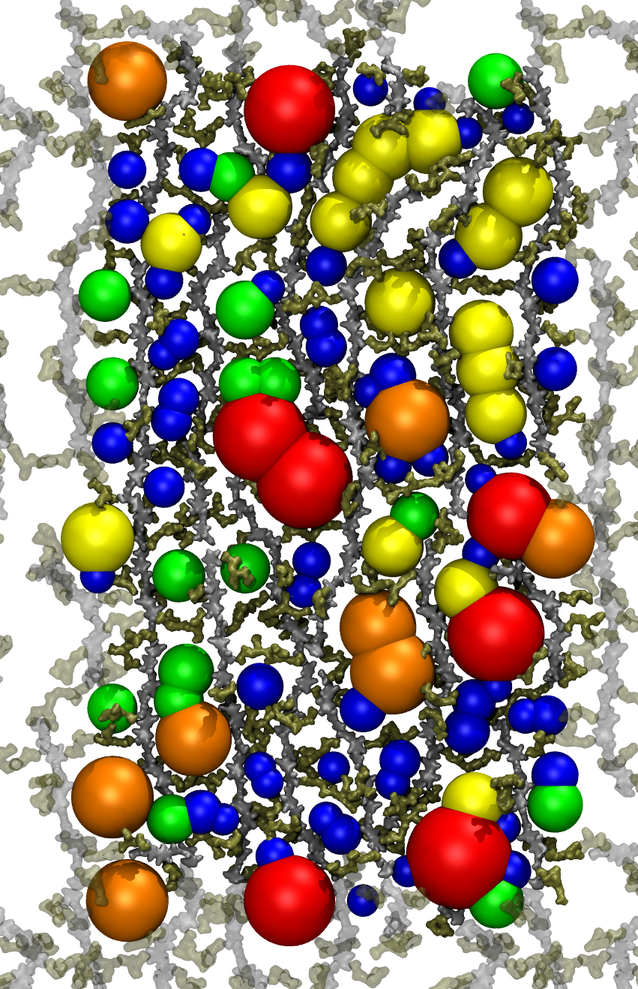

Supplement: Figure S4 — Patch of cell wall with maximum-radius spheres inscribed. Unlike in other figures, here the glycan strands are in grey and the peptides in tan. Sphere color is assigned based on size, with blue representing those with radius less than 1 nm, green less than 1.25 nm, yellow less than 1.5 nm, orange less than 1.75 nm, and red greater than 1.75 nm. (PNG) [file pcbi.1003475.s004.png]

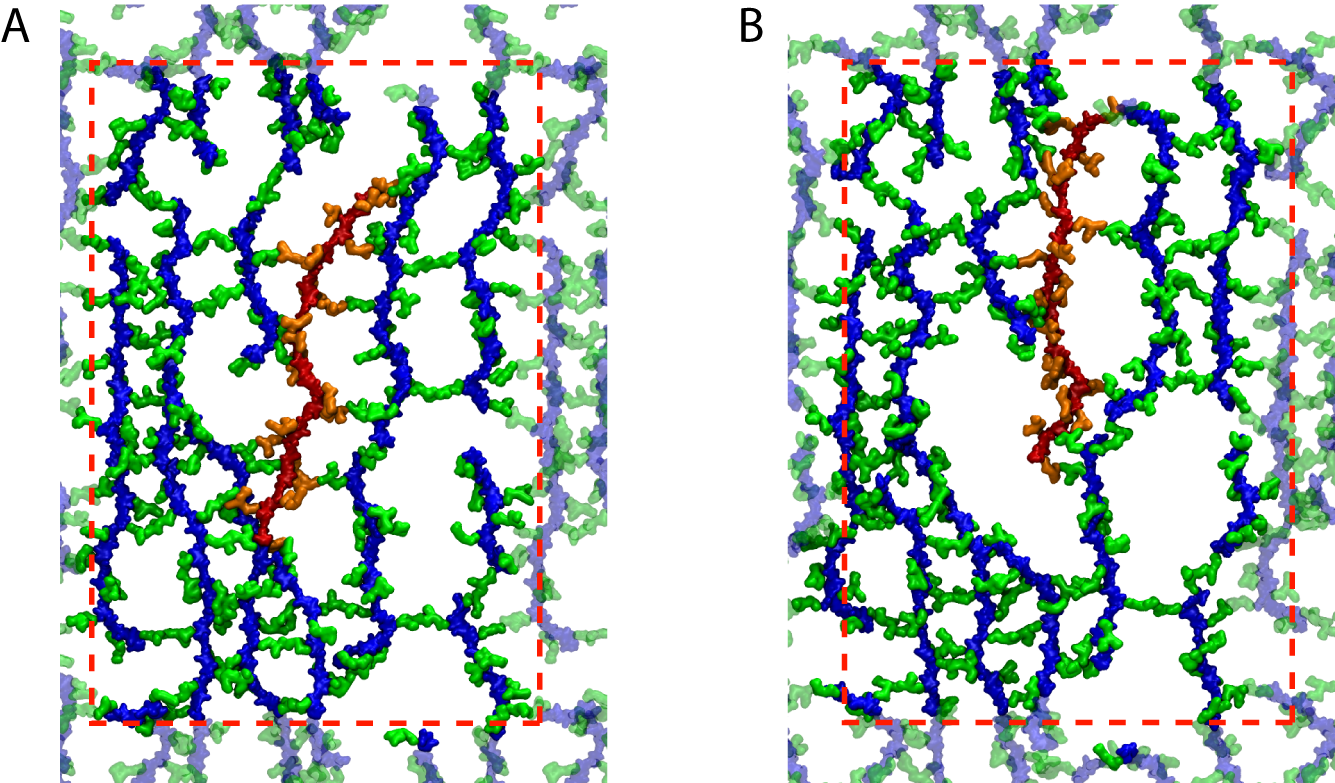

Supplement: Figure S5 — Strain-dependent insertion. In both panels, the avg17 patch is under strain . Glycan strands are in blue and peptide cross-links in green. The strand selected for deletion and later replacement is shown in red and orange. (A) Original patch. (B) Patch after strand deletion, equilibration, and subsequent strand replacement. (PNG) [file pcbi.1003475.s005.png]

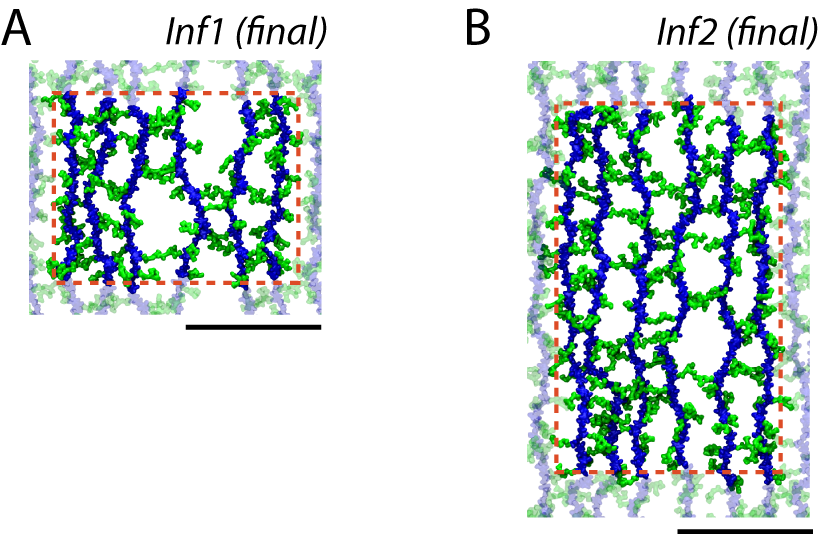

Supplement: Figure S6 — Peptidoglycan patches simulated with effectively infinite strand lengths, colored as in Fig. 3 in the main text. The black scale bars below are all equivalent at 10 nm in length. Final relaxed states for (A) inf15 and (B) inf30 are shown. (PNG) [file pcbi.1003475.s006.png]

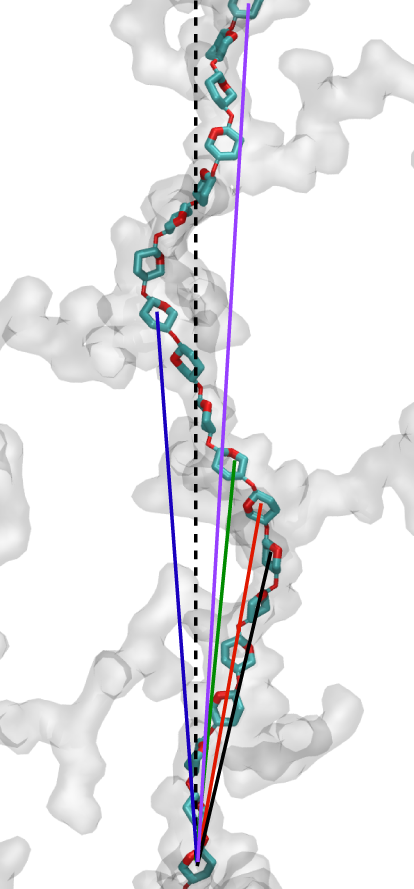

Supplement: Figure S7 — Quantifying glycan-strand angle as a measure of disorder. Shown are the NAG and NAM saccharide rings against a transparent outline of the full cell wall viewed from the outside. Individual angles made with the dashed line were measured for all vectors connecting the centers of rings spaced at least four saccharides apart, although only a subset of vectors are shown here. These vectors were then averaged over all separations within a given strand, over all strands within the simulated cell-wall patch, and over all frames in the simulation trajectory. The black, red, green, and purple vectors give positive angles, while the blue vector gives a negative angle. The dashed line represents the cell's circumferential axis with which the glycan strands were initially aligned during construction. (PNG) [file pcbi.1003475.s007.png]
