# Supplementary material for: Escherichia coli Peptidoglycan Structure and Mechanics as Predicted by Atomic-Scale Simulations
Source: PLoS Comput Biol. 2014 Feb 20;10(2):e1003475. doi: 10.1371/journal.pcbi.1003475 (PMC3930494; doi:10.1371/journal.pcbi.1003475)
Supplement: Text S1 — Formal derivation of the stress-strain relationships used in the study, expanded methods for measurements, and a discussion of simulations of strain-dependent strand insertion. (PDF) [file pcbi.1003475.s011.pdf]

Escherichia coli peptidoglycan structure and mechanics as predicted by atomic-scale simulations

James C. Gumbart[1,*], Morgan Beeby[2], Grant J. Jensen[3], and Benoît Roux[4,*]

[1] School of Physics, Georgia Institute of Technology, Atlanta, GA 30332

[2] Imperial College London, South Kensington Campus, London SW7 2AZ, UK

[3] California Institute of Technology and Howard Hughes Medical Institute, Pasadena, CA 91125

[4] Department of Biochemistry and Molecular Biology and Gordon Center for Integrative Science, The University of Chicago, Chicago, IL 60637

[*] To whom correspondence should be addressed; Email: gumbart@physics.gatech.edu, roux@uchicago.edu

Derivation of Young's moduli E_g and E_p

Under the assumption of linear elasticity, the constitutive equations for a material are known as the generalized form of Hooke's law, namely

$$\sigma_{ij} = C_{ijkm}\epsilon_{km} \tag{1}$$

where σ_{ij} is the stress tensor, ϵ_{km} the strain tensor, and C_{ijkm} is the fourth-order stiffness tensor. Although C_{ijkm} contains 81 constants, due to various symmetries not all of them are fully independent. Two additional properties of peptidoglycan further simplify the constitutive equations. First, because peptidoglycan is very thin compared to the size of a bacterium, plane stress conditions are assumed, i.e., $\sigma_{zx} = \sigma_{yz} = \sigma_{zz} = 0$. Second, peptidoglycan is orthotropic, i.e., it possesses two orthogonal planes of elastic symmetry [1]. Under these assumptions, Eq. 1, specifically its inverse, takes the form

$$\begin{bmatrix} \epsilon_{xx} \\ \epsilon_{yy} \\ 2\epsilon_{xy} \end{bmatrix} = \begin{bmatrix} 1/E_x & -\nu_{yx}/E_y & 0 \\ -\nu_{xy}/E_x & 1/E_y & 0 \\ 0 & 0 & 1/G_{xy} \end{bmatrix} \begin{bmatrix} \sigma_{xx} \\ \sigma_{yy} \\ \sigma_{xy} \end{bmatrix} \tag{2}$$

where the constants C_{ij} are now expressed in terms of traditional physical quantities, the Young's moduli $E_{x,y}$, shear modulus G_{xy} and Poisson's ratios ν_{xy} and ν_{yx}. Expanding this equation gives for the strains in the x and y directions

$$\epsilon_x = \frac{\sigma_x}{E_x} - \nu_{yx}\frac{\sigma_y}{E_y} \tag{3}$$

$$\epsilon_y = \frac{\sigma_x}{E_x} - \nu_{yx}\frac{\sigma_y}{E_y}. \tag{4}$$

By solving the equations for E_x and E_y and assigning the x axis to the peptide cross-links and the y axis to the glycan strands, the individual elasticities are determined to be

$$E_{\mathrm{g}} = \frac{\sigma_{\mathrm{g}}(1 - \nu_{\mathrm{pg}}\nu_{\mathrm{gp}})}{\epsilon_{\mathrm{g}} + \nu_{\mathrm{pg}}\epsilon_{\mathrm{p}}} \tag{5}$$

$$E_{\mathrm{p}} = \frac{\sigma_{\mathrm{p}}(1 - \nu_{\mathrm{pg}}\nu_{\mathrm{gp}})}{\epsilon_{\mathrm{p}} + \nu_{\mathrm{gp}}\epsilon_{\mathrm{g}}}, \tag{6}$$

which is precisely Eqs. 2 and 3 in the main text.

Relationships between stress and pressure

In an MD simulation, the pressures in the x, y, and z directions within the periodic simulation box can be measured and, to some degree, also controlled. In a pure medium, by virtue of Newton's first law, the pressure and the stress in a given direction are opposite one another, i.e., $P_i = -\sigma_i$ [2]. However, for simulations of the cell wall, the simulated volume is composed of both the hydrated peptidoglycan layer and a layer of water above and below. Thus, in these simulations, the pressures over the entire box, which are the quantities typically reported, can be decomposed into averages over the separate regions, the peptidoglycan layer (PG, its region given by its thickness t) and the water layers (wat., given by $L_z - t$), giving

$$\langle P_i \rangle = \int_{L_z} \mathrm{d}z P_i = \int_{L_z - t} \mathrm{d}z P_i^{\mathrm{wat.}} + \int_t \mathrm{d}z P_i^{\mathrm{PG}} = \frac{L_z - t}{L_z}\langle P_i^{\mathrm{wat.}} \rangle + \frac{t}{L_z}\langle P_i^{\mathrm{PG}} \rangle \tag{7}$$

where $i = x, y$, or z and the definition of the z-dependent pressures follows the formalism developed in the context of membrane surface tension [3, 4]. Under hydrostatic conditions, $\langle P_x^{\mathrm{wat.}} \rangle = \langle P_y^{\mathrm{wat.}} \rangle = \langle P_z^{\mathrm{wat.}} \rangle = \langle P^{\mathrm{wat.}} \rangle$. Therefore,

$$\langle P_i \rangle = \frac{L_z - t}{L_z}\langle P^{\mathrm{wat.}} \rangle + \frac{t}{L_z}\langle P_i^{\mathrm{PG}} \rangle = \frac{L_z - t}{L_z}\langle P^{\mathrm{wat.}} \rangle - \frac{t}{L_z}\sigma_i \tag{8}$$

where σ_i denotes the stress resultant (averaged over the cell-wall patch) in the i direction.

For a porous material such as the cell wall, the body force due to the water pressure can be eliminated through the definition of an effective stress, $\sigma_i' = \sigma_i + \langle P^{\mathrm{wat.}} \rangle$, for which the constitutive

relations still hold. By substituting σ'_i in Eq. 8 one obtains

$$\langle P_i \rangle = \frac{L_z - t}{L_z}\langle P^{\text{wat.}}\rangle - \frac{t}{L_z}(\sigma'_i - \langle P^{\text{wat.}}\rangle) = \langle P^{\text{wat.}}\rangle - \frac{t}{L_z}\sigma'_i, \tag{9}$$

which then can be solved for σ'_i, giving

$$\sigma'_i = \frac{L_z}{t}(\langle P^{\text{wat.}}\rangle - \langle P_i\rangle). \tag{10}$$

Finally, by solving Eq. 10 for $i = z$, $\langle P^{\text{wat.}}\rangle$ can be expressed in terms of σ'_z, giving for the effective stresses in the x and y directions

$$\sigma'_i = \frac{L_z}{t}(1 - \langle P_i\rangle) + \sigma'_z, \tag{11}$$

where $\langle P_z\rangle$ is held fixed at 1 atm in all simulations, and, under plane-stress conditions, $\sigma'_z = 0$. Eq. 11 is identical to Eq. 4 derived for simulations of microtubules in Wells and Aksimentiev [5].

The thickness t in Eq. 11 is that of the stress-bearing portion of the peptidoglycan layer and is generally not identical to that based on the mass density. To measure t in each simulation, the lateral pressure profiles in 1-Å slices along the z axis were first calculated. The resulting profiles, which are further smoothed by averaging over of a sliding 5-Å window, display a sharp increase in the lateral pressure in the direction of the applied strain. The stress-bearing region was taken to be that for which the lateral pressure is more than 10% of the maximum and the corresponding width was used as t (see Fig. S1). Due to large fluctuations in the pressure and the limited number of frames available for post-processing, we assume the magnitude of the pressure in each individual window is insufficiently converged for direct calculation of the stress [4]. Thus, we instead use Eq. 11 along with the pressure over the whole box during the original simulation, which is measured every time step rather than every frame.

Validity of plane-stress approximation

While it is common to assume a condition of plane stress in many applications, proof of its validity is often lacking [6]. To explicitly examine this assumption, and to determine an estimate for σ_z,

pressure profiles were again used. Although noise is large, there is a constant non-zero stress in the z direction. This stress is independent of the applied strains σ_{g} and σ_{p} as shown in Fig. S2, and is present even for a completely unstrained patch. Rather than arising from specific mechanical properties of peptidoglycan, however, the observed σ_z is due to an entropic pressure, i.e., the tendency of unlinked peptides to expand away from the central plane, akin to the pressure generated by an ideal gas.

The constant, but non-zero, σ_z will manifest itself in two places in the previous derivations, one for the calculation of the stresses $\sigma'_{x,y}$ from the measured pressures and one for the calculation of the elasticities from Hooke's Law. In the former case, the effect is simply additive (see Eq. 11). In the latter case, one must first consider a fully three-dimensional orthotropic material, for which the stiffness tensor becomes sixth order. Ignoring again the shear stresses, Eq. 1 becomes

$$\begin{bmatrix} \epsilon_x \\ \epsilon_y \\ \epsilon_z \end{bmatrix} = \begin{bmatrix} 1/E_x & -\nu_{yx}/E_y & -\nu_{zx}/E_z \\ -\nu_{xy}/E_x & 1/E_y & -\nu_{zy}/E_z \\ -\nu_{xz}/E_x & -\nu_{yz}/E_y & 1/E_z \end{bmatrix} \begin{bmatrix} \sigma_x \\ \sigma_y \\ \sigma_z \end{bmatrix}, \tag{12}$$

which, when multiplied out, gives for ϵ_x

$$\epsilon_x = \frac{\sigma_x}{E_x} - \nu_{yx}\frac{\sigma_y}{E_y} - \nu_{zx}\frac{\sigma_z}{E_z} \tag{13}$$

with ϵ_y and ϵ_z following similarly. Thus, the term due to σ_z can be treated simply as a perturbation on ϵ_x and ϵ_y in the equations derived for the plane-stress state. The elasticities in Eqs. 5 and 6 then become

$$E_{\mathrm{g}} = \frac{\sigma_{\mathrm{g}}(1-\nu_{\mathrm{pg}}\nu_{\mathrm{gp}})}{\epsilon_{\mathrm{g}} + \nu_{\mathrm{pg}}\epsilon_{\mathrm{p}} + f_{\mathrm{g}}(z)} \tag{14}$$

$$E_{\mathrm{p}} = \frac{\sigma_{\mathrm{p}}(1-\nu_{\mathrm{pg}}\nu_{\mathrm{gp}})}{\epsilon_{\mathrm{p}} + \nu_{\mathrm{gp}}\epsilon_{\mathrm{g}} + f_{\mathrm{p}}(z)}, \tag{15}$$

where the z dependence is accounted for by

$$f_{\mathrm{g}}(z) = \frac{\sigma_z}{E_z}(\nu_{\mathrm{zg}} + \nu_{\mathrm{pg}}\nu_{\mathrm{zp}}) \tag{16}$$

$$f_{\mathrm{p}}(z) = \frac{\sigma_z}{E_z}(\nu_{\mathrm{zp}} + \nu_{\mathrm{gp}}\nu_{\mathrm{zg}}). \tag{17}$$

We recall from Eq. 11 that the determined strains σ_{g} and σ_{p} are now also dependent on σ_z, making the final expressions for the elasticities

$$E_{\text{g}} = \frac{(\sigma_{\text{g}}^0 + \sigma_z)(1 - \nu_{\text{pg}}\nu_{\text{gp}})}{\epsilon_{\text{g}} + \nu_{\text{pg}}\epsilon_{\text{p}} + f_{\text{g}}(z)} \tag{18}$$

$$E_{\text{p}} = \frac{(\sigma_{\text{p}}^0 + \sigma_z)(1 - \nu_{\text{pg}}\nu_{\text{gp}})}{\epsilon_{\text{p}} + \nu_{\text{gp}}\epsilon_{\text{g}} + f_{\text{p}}(z)}. \tag{19}$$

where σ_{g}^0 and σ_{p}^0 denote the values under plane-stress conditions.

The elasticities are determined from the simulations by fixing one of the strains ϵ_{g} or ϵ_{p} at zero and fitting σ_{g}^0 and σ_{p}^0 as a function of the remaining strain. Re-expressing Eqs. 18 and 19 in this form gives

$$\sigma_{\text{g}}^0 = \frac{E_{\text{g}}}{(1 - \nu_{\text{pg}}\nu_{\text{gp}})}\epsilon_{\text{g}} + \frac{E_{\text{g}} f_{\text{g}}(z)}{(1 - \nu_{\text{pg}}\nu_{\text{gp}})} - \sigma_z \tag{20}$$

$$\sigma_{\text{p}}^0 = \frac{E_{\text{p}}}{(1 - \nu_{\text{pg}}\nu_{\text{gp}})}\epsilon_{\text{p}} + \frac{E_{\text{p}} f_{\text{p}}(z)}{(1 - \nu_{\text{pg}}\nu_{\text{gp}})} - \sigma_z. \tag{21}$$

Thus, although σ_z may be non-zero in our simulations, because it is independent of the applied lateral strain (see Fig. S2), it will not affect the slope of the σ^0-ϵ fit. As the elasticity is derived from this slope, the plane-stress approximation can safely be used here.

As another way to check the validity of the plane-stress approximation, we also consider the thickness of the cell-wall patch. In order to maintain an effective zero-stress state in the z direction (discounting the entropic pressure shown in Fig. S2), the thickness should respond freely to changes in lateral dimensions such that the volume remains constant [2]. More precisely, if the dimensions of the patch are given by L_x, L_y, and L_z and changes in each length by dx, dy, and dz, then

$$V = L_x L_y L_z = (L_{x,0} + dx)(L_{y,0} + dy)(L_{z,0} + dz) = L_{x,0} L_{y,0} L_{z,0}(1 + \epsilon_x)(1 + \epsilon_y)(1 + \epsilon_z) \tag{22}$$

where the strain ϵ_x is given by $dx/L_{x,0}$ (similarly for y and z). For the volume to remain constant,

$$(1 + \epsilon_x)(1 + \epsilon_y)(1 + \epsilon_z) = 1 \tag{23}$$

must be true. For the simulations run to determine E_{p} (E_x) and E_{g} (E_y), one of the dimensions was held fixed, making one of either ϵ_y or ϵ_x zero. This further simplifies the connection between the strains,

Model	$L_{z,0}$ (avg.)	$L_{z,0}$ (fit)	frac. diff.
avg8	2.72	2.91	0.070
avg17	2.27	2.09	-0.079
avg26	2.50	1.89	-0.244
Inf1	2.64	2.58	-0.023
Inf2	2.86	2.87	0.004

Table 1: Thickness under zero strain determined by averaging the weighted thicknesses measured in simulation (avg., see Eq. 25) or by extrapolating via a linear fit to $\epsilon = 0$ (fit).

leading to the equations

$$\epsilon_z = \frac{-\epsilon_i}{1+\epsilon_i} \tag{24}$$

and, thus,

$$t = L_{z,0} + dz = L_{z,0}(1+\epsilon_z) = \frac{L_{z,0}}{(1+\epsilon_i)} \tag{25}$$

where $i = x$ or y and t is the thickness of the patch.

By extrapolating the thickness as a function of the strain ϵ, the predicted thickness under plane-stress conditions, $L_{z,0}$ in Eq. 25, can be found. For each of the five cell-wall patches examined, we measured the thickness in simulation as a function of ϵ_{g} and ϵ_{p}, weighting each by $(1+\epsilon_{\text{g}})$ and $(1+\epsilon_{\text{p}})$, respectively. Should plane-stress conditions hold, these weighted values will be independent of ϵ. On the other hand, if σ_z affects the thickness in a strain-dependent fashion, extrapolation of a linear fit to $\epsilon = 0$ will provide a different value of $L_{z,0}$ than the average of the weighted thicknesses from all simulations. Table S2 compares these two metrics, i.e., the average and extrapolated values of $L_{z,0}$. For all patches except avg26, the difference between the two metrics is less than 10% and, furthermore, is not consistently positive or negative. Therefore, because the thickness in almost all cases responds freely to the imposed strain in the plane, we again conclude that plane-stress conditions are a valid approximation for the cell-wall patches simulated.

Stress-strain relationships

To calculate Young's modulus, first the slope of the stress-strain relationship was determined based on approximately 25 simulations for each constructed patch, divided between those probing E_{g} ($\epsilon_{\text{p}} = 0$) and those probing E_{p} ($\epsilon_{\text{g}} = 0$). When this slope is weighted by $(1 - \nu_{\text{pg}}\nu_{\text{gp}})$, the elasticity is recovered (see Eqs. 4 and 5 in the main text). The average stress in each simulation is plotted as a function of

strain in Fig. S3. For avg8, approximately 170 ns of simulation in total were used to determine E_{p} and E_{g} due to the slow convergence of the average pressure. For the other four systems, approximately 50 ns each was sufficient.

Calculation of pore size

In order to calculate the largest effective pore size in a given patch of peptidoglycan, first a grid of points spaced 0.3 nm apart was overlaid on the plane of the layer. At each point, the maximum-radius sphere that could be inscribed without contacting either glycan strands or cross-linked peptides was determined (see Fig. S4). Because it is assumed they are flexible and, thus, would not impede diffusion through the layer, peptides that were not cross-linked were ignored. The global maximum radius was then taken as the maximum pore size for a given structure. Although some pores are clearly irregular, experimental estimates of pore size were also calculated under the assumption that the molecules diffusing through were spherical, at least in one of two presented models [7]. Numbers reported in Table 2 in the main text are time-averaged over the course of all simulations at the stated applied strain.

Strain-dependent strand addition

To examine how strain may affect the placement of new strands in a growing cell wall, a new system was constructed. Beginning with the avg17 peptidoglycan patch under an applied strain of $\epsilon_{\text{p}} = 0.2$, one of the strands was deleted. The system was then equilibrated under tension for 10 ns, permitting the remaining strands to retract from the newly formed gap. A completely extended strand of the same length and composition as the deleted one was then added back into this gap and its glycans were held in place while the peptides were free to move for a 3 ns simulation, i.e., the same procedure used for initial construction of the entire patch. Peptides side chains that came near each other during this simulation were cross-linked, maintaining the 50% ratio of linked to unlinked as before. Finally, the resulting new patch was simulated both under constant strain ($\epsilon_{\text{p}} = 0.2$, $\epsilon_{\text{g}} = 0$) for 3 ns and also under no strain for 6 ns.

As seen in Fig. S5, some cross-links formed in new locations, resulting in a different patterning of the strands. One immediate observation is the formation of a larger pore (3.6 nm radius vs. 2.9 nm

originally), due to the elimination of a connection between the end of the re-added strand and one to its left in the figure. However, the surrounding strands do not change dramatically. When allowed to relax under zero applied tension, the new patch's equilibrium dimensions changed slightly, becoming $19.4\pm0.3 \times 32.4\pm0.3\,\mathrm{nm}^2$, as compared to $18.7\pm0.2 \times 33.4\pm0.25\,\mathrm{nm}^2$ originally. Thus, while the equilibrium area is identical (within less than 1%), the dimensions have changed by +3.7% and -3% in the peptide and glycan directions, respectively. This change is one consequence of the decrease in the diversity of strand-strand cross-links; whereas the original strand was connected to three others, the re-added one is only connected to two. Although not explicitly tested, a likely consequence of this change in cross-linking is a slightly lower E_p.

References

1. Deng Y, Sun M, Shaevitz JW (2011) Direct measurement of cell wall stress stiffening and turgor pressure in live bacterial cells. Phys Rev Lett 107: 158101-1–158101-4.

2. Ayton G, Smondyrev AM, Bardenhagen SG, McMurtry P, Voth GA (2002) Calculating the bulk modulus for a lipid bilayer with nonequilibrium molecular dynamics simulation. Biophys J 82: 1226–1238.

3. Lindahl E, Edholm O (2000) Spatial and energetic-entropic decomposition of surface tension in lipid bilayers from molecular dynamics simulations. J Chem Phys 113: 3882–3893.

4. Gullingsrud J, Schulten K (2004) Lipid bilayer pressure profiles and mechanosensitive channel gating. Biophys J 86: 3496–3509.

5. Wells DB, Aksimentiev A (2010) Mechanical properties of a complete microtubule revealed through molecular dynamics simulation. Biophys J 99: 629–637.

6. Malvern LE (1969) Introduction to the Mechanics of a Continuous Medium. Englewood Cliffs, NJ: Prentice Hall Inc.

7. Demchick P, Koch AL (1996) The permeability of the wall fabric of *Escherichia coli* and *Bacillus subtilis*. J Bacteriol 178: 768–773.
